# Improved Tissue-Based Analytical Test Methods for Orellanine, a Biomarker of *Cortinarius* Mushroom Intoxication

**DOI:** 10.3390/toxins8050158

**Published:** 2016-05-21

**Authors:** Poojya Anantharam, Dahai Shao, Paula M. Imerman, Eric Burrough, Dwayne Schrunk, Tsevelmaa Sedkhuu, Shusheng Tang, Wilson Rumbeiha

**Affiliations:** 1 Veterinary Diagnostic and Production Animal Medicine, College of Veterinary Medicine, Iowa State University, Ames, IA 50011, USA; Poojyaa@iastate.edu (P.A.); shaodahai@gmail.com (D.S.); pmartin@iastate.edu (P.M.I.); burrough@iastate.edu (E.B.); duey@iastate.edu (D.S.); 2 State Central Veterinary Laboratory, 8200 Zaisan, Khan-Uul District, Ulaanbaatar 017024, Mongolia; stsev@yahoo.com; 3 College of Veterinary Medicine, China Agricultural University, No. 2 Yuanmingyuan West Road, Haidian District, Beijing 100193, China; tangshsh@cau.edu.cn

**Keywords:** orellanine, *Cortinarius* mushrooms, renal failure, analytical method, diagnosis, liver injury, HPLC, LC-MS/MS

## Abstract

Orellanine (OR) toxin is produced by mushrooms of the genus *Cortinarius* which grow in North America and in Europe. OR poisoning is characterized by severe oliguric acute renal failure, with a mortality rate of 10%–30%. Diagnosis of OR poisoning currently hinges on a history of ingestion of *Cortinarius* mushrooms and histopathology of renal biopsies. A key step in the diagnostic approach is analysis of tissues for OR. Currently, tissue-based analytical methods for OR are nonspecific and lack sensitivity. The objectives of this study were: (1) to develop definitive HPLC and LC-MS/MS tissue-based analytical methods for OR; and (2) to investigate toxicological effects of OR in mice. The HPLC limit of quantitation was 10 µg/g. For fortification levels of 15 µg/g to 50 µg/g OR in kidney, the relative standard deviation was between 1.3% and 9.8%, and accuracy was within 1.5% to 7.1%. A matrix-matched calibration curve was reproduced in this range with a correlation coefficient (*r*) of 0.97–0.99. The limit of detection was 20 ng/g for LC-MS/MS. In OR-injected mice, kidney OR concentrations were 97 ± 51 µg/g on Day 0 and 17 ± 1 µg/g on termination Day 3. Splenic and liver injuries were novel findings in this mouse model. The new tissue-based analytical tests will improve diagnosis of OR poisoning, while the mouse model has yielded new data advancing knowledge on OR-induced pathology. The new tissue-based analytical tests will improve diagnosis of OR poisoning, while the mouse model has yielded new data advancing knowledge on OR-induced pathology.

## 1. Introduction

Orellanine is a potent natural bipyridyl toxin produced by *Cortinarius* mushrooms, which grow throughout Europe and parts of North America. *Cortinarius orellanus* and *C. rubellus* are the major orellanine-containing mushrooms which are responsible for causing human intoxications. In these mushrooms, orellanine occurs mainly in form of its mono- and diglucoside [1].

The toxicity of *Cortinarius* mushrooms was first recognized in 1957 in Poland [2]. About 102 individuals were intoxicated by ingesting *C. orellanus*, 10% of whom died [2,3]. Acute renal failure (ARF) is the major presentation of *Cortinarius* human mushroom poisoning, but liver injury has also been reported [2,4]. Recently *C. orellanosus* was reported to have been responsible for a case of human poisoning in Michigan [5].

Acute renal failure typically develops 7–14 days post ingestion of the poisonous mushrooms, but can occur as early as 3 days after. The hepato-renal syndrome reported in some human cases is characterized by increased serum liver enzymes and bilirubin [6]. Histologically, human lesions include tubulo-interstitial nephritis and liver cellular necrosis and lipidosis [2]. Other clinical signs reported in *Cortinarius* mushroom intoxications include headache, gastrointestinal distress and neuromuscular symptoms such as limb paresthesia and myalgia [6]. In veterinary medicine, the first case of *Cortinarius* mushroom poisoning in animals was reported in sheep [7]. The disease, which was confirmed by feeding the mushrooms to sheep, was characterized by hypocalcemia, hyponatremia, proteinuria, and hemoglobinuria. Grossly, petechial hemorrhages in the skin, subcutis, and epicardium were observed while tubulointerstitial nephritis was seen histologically [7]. There are many other toxin-induced causes of ARF in humans and in animals, including arsenic, inorganic mercury, cadmium and lead; natural toxins such as ochratoxins, aflatoxins, and citrinin; medications; and other mushroom species such as *Amanita smithiana* [8,9,10]. Therefore, the diagnosis of *Cortinarius* mushroom intoxication cannot be made on the basis of clinical presentation alone.

A conclusive diagnosis of any human and animal intoxication rests on many pillars, including a history consistent with presence of toxin in the environment or food; clinical signs consistent with the suspect toxin; a complete blood cell count (CBC) and a comprehensive serum chemistry and urinalysis profile; gross and histological findings consistent with the suspect toxin; and especially analytical confirmation of the presence of the toxin and/or its metabolites in biological fluids and tissues. Identification and quantitation of OR in tissues is a critical step in forensic and clinical diagnostic investigations of *Cortinarius* mushroom intoxication.

A comprehensive review of the literature revealed a lack of suitable definitive tissue-based analytical test methods for quantifying OR in tissues. Existing tissue-based analytical tests are difficult to perform; most are based on outdated technologies like thin layer chromatography (TLC) and electrophoresis which are riddled with false positives and false negatives, and lack sensitivity and selectivity. This poses challenges to forensic and diagnostic communities because the use of such procedures is likely to yield unreliable results with false conclusions.

Rapior *et al.* used TLC to analyze for OR and its breakdown product orelline in human renal biopsies [11]. TLC is nonspecific because it is subject to cross reactivity with non-target molecules. Results were reported as OR “equivalents” using unconventional units. Orellanine “equivalent” concentrations in renal biopsies were reported as 7 µg/25 mm^3^ on Day 13 post ingestion of mushrooms and 24 µg/8 mm^3^ at 6 months post ingestion. These results are difficult to translate to conventional units, such as parts-per-million or µg/g commonly used for forensic toxicological and diagnostic interpretations. Rohrmoser *et al.* also used TLC to analyze human body fluids, dialysis fluids, and kidney biopsies for OR and orelline [12]. Kidney OR concentration was reported as 160 ng/µL and 35 ng/µL for samples taken on a few days apart [12]. These unconventional units, ng/µL for tissues, provide challenges in interpretation of toxicological data. Rohrmoser *et al.* [12] also questioned the validity of results of one study by Andary *et al.* [13], who analyzed renal biopsies using high performance liquid chromatography (HPLC) and TLC techniques. HPLC and TLC are not confirmatory procedures.

Holmdahl *et al.* [14] did preliminary studies of OR analysis in serum and kidney using HPLC equipped with an electrochemical detector. In developing their serum and kidney assay they spiked serum and kidneys of untreated mice with OR. Orellanine added to serum was completely recovered, but the recoveries of the kidney spikes were very poor, at only 25%. These investigators did not report any serum or kidney OR concentration in their animal toxicity studies, but reported results of OR in mushrooms only. For the kidney, they reported histopathology results only and no quantitative OR data was reported [14]. Another limitation of their mouse study was the lack of CBC, urinalysis data, and the histopathology of other organs [14].

Herrmann *et al.* developed a method based on quadrupole time-of-flight mass spectrometry/mass spectrometry (QTOF-MS/MS) for analysis of OR in blood and mushroom stew [15]. An extensive description of extraction of OR from the mushroom was provided. Studies of rat blood spiked with OR recovered 60% of the added OR. The limitation of that comprehensive study was that the method for kidney OR analysis was not described [15]. Since the kidney is a more complex matrix than serum, the serum method cannot be directly transferred to analyze the kidney without further optimization. This is because OR is known to be tightly bound in the kidney and extraction from the kidney is more complex than in serum or mushrooms [12]. The OR in the kidney is reportedly insoluble and cannot be removed by dialysis [12]. A second limitation of their study was that they did not extend their method to analyze tissues or serum from animals naturally intoxicated or experimentally dosed with either OR or *Cortinarius* mushrooms. Thus the performance of the method for detection of OR in animals naturally poisoned by *Cortinarius* mushrooms could not be evaluated.

On the basis of this background, it was apparent that more research on tissue-based analytical test methods for forensic purposes and diagnosis of OR intoxications in humans and animals was needed. We therefore conducted this study with two specific objectives: (1) to develop definitive quantitative HPLC and liquid chromatography/mass spectrometry (LC-MS/MS) tissue-based analytical methods for OR and (2) to investigate morphological, blood and serum biochemical changes, urine changes, and multiple organ histological changes in mice injected with OR. We chose to work with mice because there are few studies in this species and also due to the limited amount of OR we had at hand, the small size of the mouse was appealing.

Overall, the value of this study was to improve the diagnosis of OR poisoning in humans and animals using tissue-based diagnostic tests, CBC and clinical chemistry, histopathology, and urinalysis using a less commonly used animal model, the mouse.

## 2. Results

### 2.1. Clinical Observations

**Figure 1:**
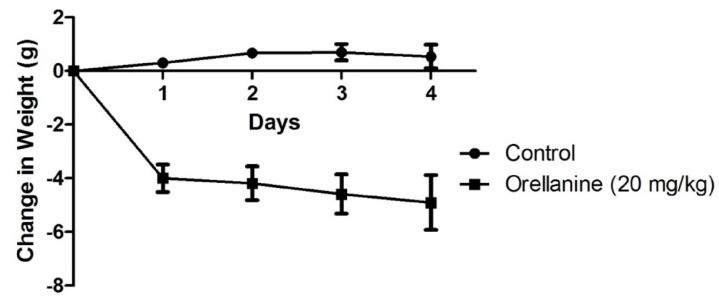
Change in body weight in mice given orellanine compound to those given 0.9% normal saline. Orellanine caused a significant reduction in body weight compared to controls (*p* < 0.01).

**Figure 2:**
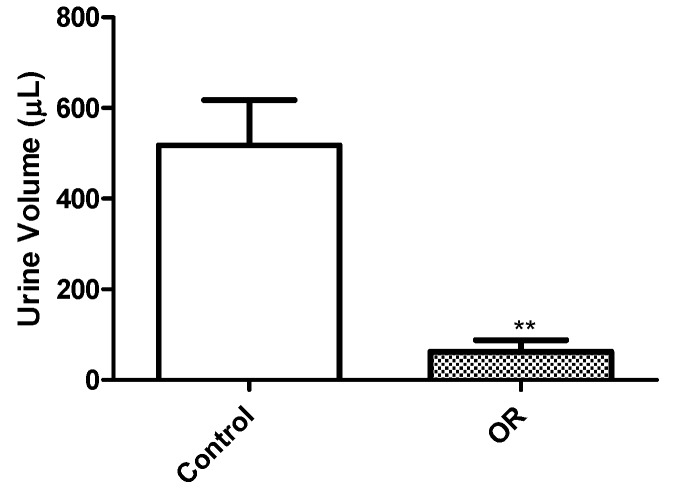
Total urine volume in mice given orellanine or 0.9% normal saline. Represents five mice per group over 12 h before euthanasia at 96 h. Urine output was significantly reduced in mice given orellanine compared to controls (** *p* < 0.01).

Negative control mice injected with saline did not exhibit any clinical signs during the course of the study. However, mice given OR exhibited clinical signs starting 24 h after OR injections, but clinical signs became more prominent after 48 h. One mouse from the OR-treated group was euthanized 72 h after OR dose because it was moribund. The most significant clinical observations in OR-dosed mice included loss of appetite, lethargy, loss in body weight, and oliguria. The OR-dosed mice also looked dehydrated as evidenced by changes in skin elasticity. Changes in body weight are shown in Figure 1, while changes in urine output on the day before the 96 h euthanasia is shown in Figure 2. On Day 4 (96 h), most OR-dosed mice exhibited piloerection and appeared rounded, were ataxic when mobile and were most often sedentary.

### 2.2. Gross Observations and Organ Weight Changes

Kidneys of OR-dosed mice were pale relative to controls, especially those from mice euthanized at 96 h. Stomachs of OR dosed mice were grossly distended with gas and the fecal pellets in the large intestines were dark. The spleens of OR-treated mice were grossly reduced in size and weight starting from 24 h post OR exposure and onwards. Changes in organ weight are shown in Table 1. There were no changes in kidney weights of OR-dosed mice compared to controls. However, there was a statistically significant reduction in liver weights of mice given OR compared to saline control mice, starting 2 h post OR exposure. Lung weights of OR dosed mice were significantly reduced 2 h post-OR exposure only.

### 2.3. CBC and Serum Chemistry Profile Results

Changes in CBC are summarized in Table 2. Although there were no statistically significant changes in total white blood cell counts, OR caused a significant increase in total neutrophil counts and a statistically significant reduction in total lymphocyte and eosinophil counts. The neutrophilia was noticeable starting 24 h post OR exposure, whereas lymphopenia and eosinophilia were immediate, starting at 2 h post-OR exposure. Orellanine also caused a significant reduction in total platelet counts. Platelet counts were reduced by about 71% from a control mean 1161 × 10^3^/µL to about 333 × 10^3^/µL, 96 h post OR exposure. There were no statistically significant changes in total red blood cell counts, total hemoglobin, or hematocrit, but OR caused a statistically significant reduction in mean corpuscular volume (MCV) and a statistically significant increase in mean corpuscular hemoglobin concentration (MCHC) 96 h post OR-exposure. Presence of nucleated red blood cells (RBC) was observed, but only at 96 h post OR exposure.

A summary of serum biochemical changes in OR-treated mice is provided in Table 3. Unfortunately, because most OR-dosed mice were dehydrated at the 96 h time point, sufficient blood to run for all tests was obtained from 3 mice only. There was a statistically significant increase in blood urea nitrogen (BUN), alanine aminotransferase (ALT) and alkaline phosphatase (ALP) 96 h post OR exposure. These serum results suggest there were both renal and hepatic injuries in mice injected with OR. Although there were no statistically significant changes in total serum protein, serum albumin concentration was significantly reduced at 2 and 24 h post-OR exposure. However, serum albumin concentration appeared to rebound to normal range at 96 h. Serum electrolyte changes in mice given OR were characterized by hypocalcemia, reduced sodium ion concentration, and a significant elevation in serum phosphorus concentration (Table 3).

With regard to urinalysis results, unfortunately because of oliguria, we were only able to perform qualitative tests because of inadequate urine sample volume which precluded extensive urinalysis testing. Urine from OR dosed mice tested positive for hematuria and proteinuria (2+) and was slightly acidic with a pH in the range of 5.5–7.0. On the contrary, urine from control mice tested negative for blood, and proteins and the pH was in the 7.0–8.0 range. Urine from OR dosed mice was pale yellow while that of controls was yellow. There was no change in urine specific gravity.

### 2.4. Histopathology Results

**Figure 3:**
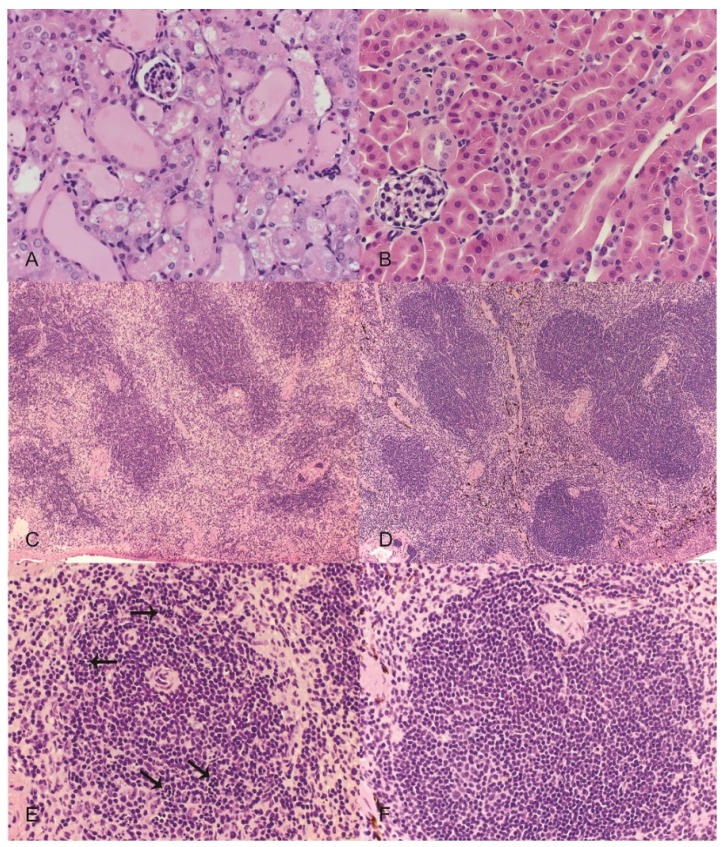
Representative images from mice treated with orellanine (OR) or saline controls (CON). Hematoxylin and eosin. (**A**) (top left) Kidney from an OR-treated mouse revealing variable tubular degeneration and ectasia with luminal accumulations of proteinic fluid and occasional sloughed cells; (**B**) (top right) kidney from a CON mouse with normal tubular morphology; (**C**) (middle left) spleen from an OR-treated mouse revealing an overall reduction in red and white pulp cellularity; (**D**) (middle right) spleen from a CON mouse with normal cellularity and well-defined red and white pulp margins; (**E**) (bottom left) higher magnification of white pulp from an OR-treated mouse revealing numerous pyknotic and karyorrhectic lymphocytes (arrows); (**F**) (bottom right) higher magnification of white pulp from a CON mouse with normal morphology. Scales: A,B,E,F = 600× magnification; C,D = 100× magnification.

**Figure 4:**
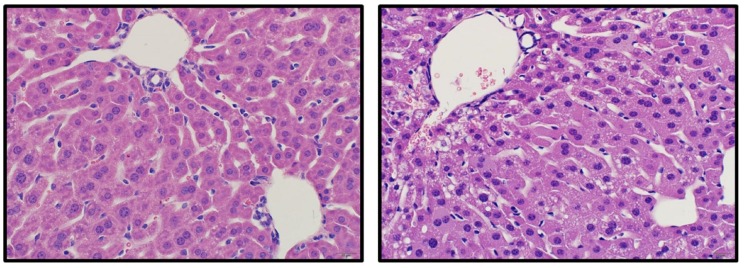
An image from a control liver (**left**) and a mouse (**right**) that exhibited hepatocellular vacuolation following exposure to OR. Scale: 400× magnification.

Significant lesions were observed in the renal cortex of all examined OR treated mice and included variable degeneration and necrosis of the tubular epithelium with regeneration, tubular ectasia, and tubular proteinosis (Figure 3A). Affected tubules often contained low numbers of sloughed epithelial cells. The renal tubules of control mice were unremarkable (Figure 3B). The spleens of OR treated mice were smaller with reduced overall cellularity relative to control mice (Figure 3C,D) and the white pulp of OR treated mice was characterized by moderate to high numbers (10%–50%) of pyknotic and karyorrhectic lymphocytes (Figure 3E,F). Moderate random hepatocellular vacuolation was observed in one OR-treated mouse (Figure 4). Lesions were also observed in the stomachs of mice given OR and included presence of neutrophils within the lamina propria of the non-glandular stomach with variable associated thickening of the squamous epithelium and submucosal edema; however, low numbers of neutrophils were also occasionally observed in the lamina propria of control mice at the junction of the glandular and non-glandular stomach.

### 2.5. HPLC Method Validation and Analysis of OR

**Figure 5:**
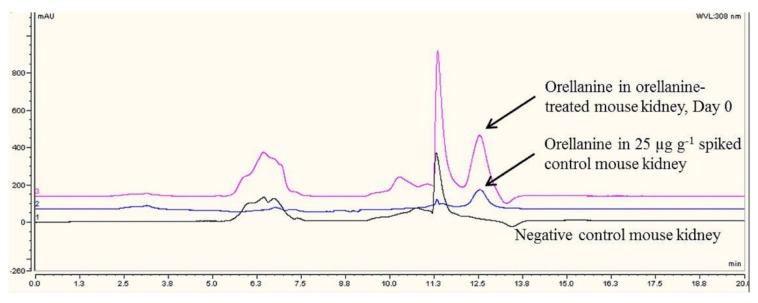
(From top to bottom) HPLC chromatographs of orellanine in an orellanine-exposed mouse kidney (Day 0), an orellanine-spiked control mouse kidney (25 µg/g), and a negative control mouse kidney.

HPLC chromatographs of OR were shown in Figure 5 for an OR-treated mouse kidney. Results of mouse kidneys fortified at different concentrations of OR, are shown in Table 4. Three calibration curves were established on three separate runs. Mouse kidney samples were fortified with OR at concentrations in the range of 10–50 µg/g. This analytical range was chosen because it is clinically relevant. In human OR intoxication cases, kidney biopsy OR concentrations were found to be in the range of 35–160 µg/mL [12]. The correlation coefficients (*r*) were excellent and generally above 0.99 except for the first one which was 0.97. The method limit of quantitation (LOQ) was 10 µg/g.

Comparing the nominal concentrations from all three calibration curves, we calculated the inter-day repeatability in terms of relative standard deviation (RSD), as shown in Table 5. The RSD varied from 1.3%–9.8% for OR tissue concentration range of 15–50 µg/g, indicating the good repeatability of the method. Accuracy was reported as the relative difference between the back-calculated concentration and fortification levels. The results were between 1.5% and 7.1% for the range of 15–50 µg/g. Therefore these results supported the validity of the calibration curves and sample preparation procedure.

Following validation of the HPLC method, we proceeded to analyze the kidneys of mice injected subcutaneously (SC) with OR for presence of OR. The results are shown in Table 6. Orellanine was only found in the kidneys of mice given OR. The highest OR concentration (97 ± 51 µg/g) was found 2 h post OR-exposure. The kidney OR concentration gradually declined, but was still present when the experiment was terminated at 96 h post OR exposure (17 ± 1 µg/g).

### 2.6. Results for Liquid Chromatography-Mass Spectrometry/Mass Spectrometry (LC-MS/MS)

**Figure 6:**
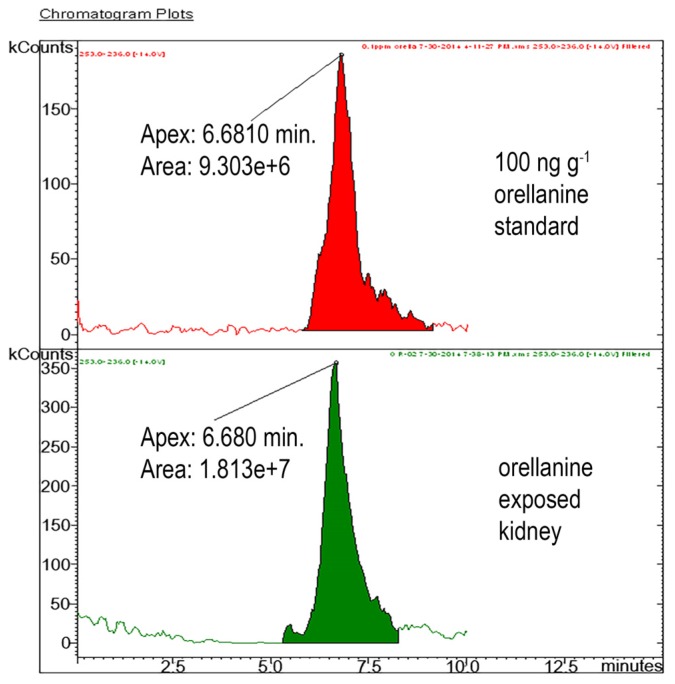
ESI-LC-MS/MS chromatograms for transition peak 253 > 236 of 100 ng/g orellanine standard (upper graph) and orellanine in exposed kidney (lower graph).

Evaluation of the LC-MS/MS method for kidneys showed an average OR recovery of 91%. The LOD was 20 ng/g. We then proceeded to analyze kidneys for presence of OR. Representative chromatographs of OR in solvent (upper graph) and from renal tissue of mice exposed *in vivo* (lower graph) are shown in Figure 6. Orellanine was only found in the kidneys of mice given OR. LC-MS/MS results give a confirmatory response for the presence of OR in the kidney tissue. This demonstration of OR using triple quadrupole LC-MS/MS using incurred OR in tissue shows definitive confirmation over spiked samples shown by Herrmann *et al.* [15].

## 3. Discussion

There is a substantial body of published literature on *Cortinarius* mushroom poisoning in humans in Europe and North America which is partly covered in reviews [6,16]. *Cortinarius* mushroom poisoning has also been reported in animals [7]. Orellanine is the active nephrotoxin in *Cortinarius* mushrooms [1,17].

A comprehensive review of the literature revealed that a complete toxicity profile of *Cortinarius* mushrooms has not yet been established. A comprehensive toxicity profile is essential for proper diagnosis and treatment of OR poisoning. Most rodent studies to-date have been narrow in scope, more or less focusing on renal effects of *Cortinarius* mushroom intoxications [3]. Another related challenge is that most rodent studies were conducted with rats, yet some reports suggest rats are not very sensitive to OR poisoning [3]. The few studies that utilized mice were limited in scope, and the kidney, other organs such as the immune system, the liver, and lungs were not examined histologically.

In this study, we took a comprehensive approach to understand the toxicity of OR in mice. Results confirm that the kidney is a target organ of OR poisoning as shown by histological changes of proximal tubular necrosis and supporting serum chemistry profiles, indicative of ARF, including elevated BUN and serum phosphorus. Although there was no statistically significant increase in serum creatinine, the trend in this acute short term study was one of increased serum creatinine at the time the study was terminated at 96 h. However, this is the first report of renal weight in animals experimentally dosed with OR or *Cortinarius* mushrooms. It is interesting that there were no significant changes in renal weight of mice given OR. Frequently, intoxications leading to ARF, such as that caused by mercuric chloride, are characterized by increased renal weight [9]. Renal weight increase is often associated with interstitial edema, which was not observed in the present study.

The proteinuria and hematuria observed in these mice is likely of tubular origin because histopathology showed tubular necrosis. Urine of mice given OR was acidic, but the cause of this is not clear. However, because mice were anorexic, it is probable that ketosis contributed to low urine pH observed in mice in this study. Ketonuria should be evaluated in future studies.

This comprehensive OR mouse study has demonstrated that other organs besides the kidney are affected. In particular, liver injury was demonstrated by reduced liver weight, elevated liver enzymes, and histopathology characterized by mild hepatocellular vacuolation. This finding is consistent with reported hepato-renal syndrome in some human cases of *Cortinarius* mushroom intoxication [17]. The reasons for reduced liver weight are unknown. A possible hypothesis is that mobilization of glycogen and or/lipids because of anorexia caused reduced liver weight. Of particular note, hepatotoxicity has not been reported in rats, the most widely used rodent model of OR or *Cortinarius* mushroom intoxication. This is interesting because it is suggested in the literature that rats are more resistant to OR poisoning than humans [3]. It is possible that interspecies differences in sensitivity to OR exist and that mice are more sensitive to OR than rats and thus a more suitable animal model for human disease.

A novel finding revealed by the present study is splenic atrophy, characterized by lymphocytolysis. We also observed significant changes in white blood cell counts including neutrophilia, lymphopenia and eosinopenia. Whether these leukocyte changes can be explained by stress and corticosteroid induced immunosuppression remains to be determined in future studies.

Another interesting observation from this study was thrombocytopenia. Thrombocytopenia may cause hemorrhage. Overås *et al.* reported petechial hemorrhages in the skin, subcutis and subepicardially in lambs poisoned after natural ingestion of *Cortinarius* mushrooms [7]. Unfortunately, a CBC was not reported in that case report. It is also interesting however that Overås *et al.*, like in the present study, also reported a pronounced hypocalcemia and hyponatremia in sheep which presumably died of *C. speciosissimus* poisoning [7]. These results underscore the importance of including a CBC as part of a clinical evaluation of humans and animals suspected to have ingested *Cortinarius* mushrooms. Hematological changes consistent with those reported here are suggestive of *Cortinarius* mushroom poisoning. This is particularly important in veterinary medicine where history of mushroom ingestion is sometimes vague and incomplete.

Gastrointestinal effects are among the earliest effects noted in human victims of OR poisoning. These include abdominal pain, vomiting, and diarrhea which may revert to constipation. These effects appear 2–3 days post ingestion and there is no explanation given regarding their origin. In this study, gastrointestinal effects were seen in mice even though OR exposure was by SC injection. Mice given OR had bloated stomachs and the fecal content in the lower small intestines and in the large intestines were dark, suggestive of melena. However, histopathology of the gastrointestinal tract (GIT) did not reveal epithelial necrosis and the reason for dark feces presently is unknown. The only histological changes noted in the GIT were in the stomach and were characterized by neutrophil infiltration of lamina propria of the glandular stomach. Unlike bloating which was present in all OR-dosed mice, these lesions were not universally present and the significance of these finding remains unclear at the present time.

The limitations of currently published methods for analysis of OR in tissues were presented in the introduction. We now have developed tissue-based diagnostic analytical test methods for diagnosis of OR poisoning. We chose the kidney as a sample of choice based on case reports in the human literature suggesting the kidney is the ideal specimen for diagnosis of OR poisoning for up to 60 days after clinical signs of OR poisoning manifest [12]. A reliable, sensitive, and quantitative HPLC method for quantitation of OR has been established in our laboratory. We have used this method to quantify OR, the biomarker of *Cortinarius* mushroom intoxication in kidneys of mice dosed with OR. Our results have shown that in mice dosed with a toxic dose of OR approximating the mouse LD_50_, the concentration of OR in the kidney was a mean of about 97 µg/g (parts-per-million, ppm) soon after injection, but it declined to a mean of 17 ppm 96 h post-injection. This represents an 82% reduction in kidney orellanine concentration over 96 h. In this study, we only analyzed for orellanine but not orelline, which has been reported in studies using TLC [12]. Our results suggest that the concentration of OR substantially declines in the kidney over a 48 h time period, perhaps due to metabolism and/or elimination through urine. Orellanine has not been found in urine after 48 h in some human case reports [12].

To be absolutely certain that we were specifically measuring OR, we established a confirmatory LC-MS/MS analytical test for OR. As expected, the LC-MS/MS method was more sensitive than the HPLC method. The LC-MS/MS is specific for OR because it detects specific daughter ions of the parent OR compound. We recommend using HPLC for routine analysis and the LC-MS/MS for unequivocal confirmation of the presence OR in the kidney. Results of this study showed that the HPLC method is sensitive enough to detect diagnostically relevant kidney OR concentrations which are expected to be > 10 ppm (10 µg/g tissue), which have been reported for human cases [12]. The other advantage is that HPLC is also commonly available in most diagnostic laboratories compared to LC-MS/MS. Our veterinary diagnostic laboratory is the only laboratory to have established tissue-based diagnostic tests for OR in the North America. Using these diagnostic tests, we have shown that OR is present only in the kidneys of mice given OR. Orellanine was not present in control mice. Our results also show that kidney OR concentrations > 10 ppm are diagnostic of OR poisoning.

## 4. Experimental Section

### 4.1. Animal Studies

The primary objective of the animal study was to expose mice to OR *in vivo* and collect biological samples from OR-dosed and control mice for diagnosis of OR poisoning. This was not primarily a study of OR toxicity in mice, but rather the objective was to use tissues and fluids from exposed animals to develop a comprehensive diagnostic profile of OR poisoning in the mouse. All animal studies were approved by the Iowa State University Institutional Animal Care and Use Committee (IACUC) (4-12-7341-M, 30 March 2014). The C57/BL6 (6–7 weeks) male mice used in these studies were purchased from The Jackson Laboratories (Bar Harbor, ME, USA). They were housed 4 per cage in the Laboratory Animal Resource (LAR) Facility of the Iowa State University College of Veterinary Medicine (ISU CVM). The cage contained environmental enrichment appropriate for mice. The mice were acclimated for 1 week to a room temperature of 20–21 °C, relative humidity of 35%–50% and a 12 h light cycle. Light automatically turned on at 6 AM and turned off at 6 PM. They were provided 14% Protein Rodent maintenance diet (Teklad Global, Harlan Sprague Daley Inc., Indianapolis, IN, USA) and drinking water *ad libitum*. Environmental enrichment in the form of toys appropriate for mice were provided 24/7.

Mice were randomly assigned to one of the following two study groups, 12 mice per group: A negative control group of mice received 0.9% normal saline at 20 mL/kg body weight (bw) subcutaneously (SC) on Day 0; test mice were injected 20 mg/kg bw OR SC on Day 0. Subsets of mice given OR were euthanized 2 h, 24 h, or 96 h after OR injection. The OR dosage was selected basing on intraperitoneal (IP) LD_50_ values reported for mice [6]. The objective was to use a dosage that would certainly cause nephrotoxicity in mice so that we could sufficiently evaluate diagnostic criteria for OR intoxication in mice. We chose the SC over the IP route because the former is less invasive. Also, given the limited supply of OR at hand, we preferred the SC route over oral administration, as the IP route would require more toxin. Ultimately, the SC route of administration did not impact the primary objective of the study, which was to expose mice to OR and collect biological samples from OR-dosed and control mice for diagnosis of OR poisoning.

Injectable OR solution was prepared by dissolving 3 mg of OR in 150 µL pyridine (Sigma Aldrich, St. Louis, MO, USA) and bringing the total volume to 3mL with 0.9% normal saline. The final amount of pyridine in the injected solution was only 5%. In order to minimize the number of mice for this study, negative control mice injected with 0.9% normal saline were euthanized at 96 h, the same time point as the longest held OR dosed mice in this study.

Mice were weighed on Day 0 prior to treatment, and daily thereafter until euthanasia. Mice were examined daily and any observed clinical signs were recorded. Pooled urine was collected from four test mice and four negative control mice placed in wire bottom cages over a 12 h time period, starting immediately after dosing on Day 0 and on the day before euthanasia. The urine was collected from the bottom of plastic cages every 3 h during the 12 h collection period using a syringe to avoid evaporation. Urine volume was determined using a graduated syringe. Urine samples were refrigerated before submission for urinalysis testing in the Clinical Pathology Laboratory of the Iowa State University College of Veterinary Medicine (ISU CVM). Urinalysis was done qualitatively using manual dipsticks, visually for color, or microscopically for sediments. Parameters evaluated included color, transparency, specific gravity, glucose, bilirubin, ketones, blood, pH, protein, casts, white and red blood cells, crystals, bacteria, and presence of epithelial cells. Unfortunately, the sample size of urine collected from dehydrated OR-dosed mice precluded analysis of urine for OR.

Mice designated for a CBC were euthanized using carbon dioxide by inhalation. Blood was collected from the heart using an 18 gauge needle and either placed in ethylenediaminetetraacetic acid (EDTA) tubes for a CBC or in non-anticoagulated red top tubes, allowed to clot, and then centrifuged to collect serum for serum chemistry profile analysis. Both the CBC (*n* = 5/group) and serum chemistry profiles (*n* = 11/group) were performed in the Clinical Pathology Laboratory, ISU CVM. The hematology and CBC were performed on a hematology system and the serum chemistry profile was run on an Abaxis analyzer (VetScand, Abaxis, Union City, CA, USA). The hematology consisted of total and differential while blood cell counts. The serum chemistry profile included total protein, albumin, alkaline phosphatase (ALP), alanine transferase (ALT), amylase, total bilirubin, blood urea nitrogen (BUN), calcium, phosphorus, creatinine, glucose, sodium, and potassium. Unfortunately, serum sample volume was insufficient, which precluded running OR.

Various tissues including the kidneys, liver, spleen, and lungs were collected, weighed, and either frozen at −80 °C until analysis for OR analysis. We chose the kidney (*n* = 6) for OR analysis because it is the primary target organ and is the sample of choice for OR analysis [12]. Mice designated for histopathology (*n* = 3 per group) were anesthetized with a ketamine/xylazine combination (90 mg/10 mg)/kg IP. Once anesthetized, the thoracic cavity was opened to expose the heart. Phosphate buffered saline (PBS) (Sigma Aldrich, St. Louis, MO, USA) was injected through the left heart ventricle followed by 4% paraformaldehyde (Sigma Aldrich, St. Louis, MO, USA). Upon full perfusion, samples collected for histologic examination included lung, liver, kidney, spleen, heart, stomach, duodenum, jejunum, ileum, cecum, and colon. Samples were placed in 10% neutral-buffered formalin for at least 24 h, embedded in paraffin, sectioned at 4 µm and processed routinely for hematoxylin and eosin (H&E) staining.

### 4.2. Chemicals and Reagents for Chromatography Analysis of OR

HPLC-grade methanol, HPLC-grade acetonitrile, concentrated hydrochloric acid, ammonium hydroxide, ammonium acetate, *o*-phosphoric acid (85%), formic acid, and 0.45 µm nylon syringe filters were purchased from Thermo Fisher Scientific (Waltham, MA, USA). Orellanine standard (95%) and for animal experiments was purchased from Ramidus AB (Lund, Scania, Sweden). All aqueous solutions were prepared in 18.2 MΩ·cm water (Aries Filter Network, West Berlin, NJ, USA). Bond Elut Jr mycotoxin columns (Agilent Technologies, Santa Clara, CA, USA) were purchased for clean-up columns. A 3M hydrochloric acid solution was prepared by adding 24.9 mL concentrated hydrochloric acid into pure water and bringing the volume to 100 mL with water. A 3M hydrochloric acid in methanol solution was prepared by adding 24.9 mL concentrated hydrochloric acid into HPLC-grade methanol and bringing the volume to 100 mL by methanol. A 4 mM ammonium acetate aqueous solution was prepared by dissolving 0.308 g ammonium acetate in pure water and bringing the volume to 1 L. A 1% formic acid aqueous solution was prepared by diluting 5 mL formic acid to a total volume of 500 mL with water. Orellanine standards were prepared by dissolving corresponding amounts of OR pure standard in hydrochloric acid solution (pH 1.5). Because OR is known to photodegrade, the OR standards were stored in aluminum-foil-wrapped vials for protection from light.

### 4.3. Sample Preparation for OR Analysis by HPLC

We focused on analyzing the kidney because published literature of intoxication in humans suggests it is the tissue of choice for OR testing [12]. Whereas OR was not detectable in serum and urine after onset of symptoms, it was detectable in renal biopsies taken 60 days after onset of intoxication [12]. We chose to include HPLC in our study because this analytical platform is commonly available in many veterinary diagnostic toxicology laboratories. To start, an in-house HPLC method was developed to analyze for OR in the kidney. The tissue sample preparation procedure was a significant modification from a method reported by Herrmann *et al.* [15]. As indicated in the introduction, the limitations of Herrmann [15] study were: their method focused on analysis of mushrooms; kidney OR analysis was not described; the mushroom method was not applied to analysis of tissues or serum derived from animals dosed either with OR or *Cortinarius* mushrooms; and they used QTOF-MS/MS, an expensive platform found only in a few diagnostic laboratories. As described, the method was not ideal for analysis of OR in the kidney. Three major changes were made: (a) A clean-up step was added to further remove the interferences from OR. This cleanup step gave good selectivity for OR. (b) We discovered that the pH of mobile phases was critical in stabilizing OR in the HPLC analysis, we replaced formic acid with o-phosphoric acid because the latter is less volatile and is able to maintain the pH for an extended period of time. (c) A more time-efficient extraction step combining vortex mixing and sonication was introduced, to replace the original 3-h extraction procedure for mushrooms. These major changes tailored to kidney extraction yielded good selectivity and sensitivity.

Briefly, approximately 0.1 g mouse tissue was homogenized in 4 mL 3M hydrochloric acid in a vortex mixer for 5 min at 2500 rpm. The mixture was then sonicated for 20 min at room temperature, followed by centrifugation at 3000 rpm (1744× *g*) for 30 min. The supernatant was neutralized with 1 mL ammonium hydroxide to adjust to the optimum pH for clean-up, and then loaded onto clean-up columns. After washing the columns with 20 mL pure water followed by 20 mL methanol (both adjusted to pH 1.5 with formic acid), the OR was eluted with 5 mL 3M hydrochloric acid in methanol at a flow rate of 10–20 mL/min. The eluent was concentrated to 0.2 mL under nitrogen.

### 4.4. HPLC Conditions

Measurements were performed with an HPLC system equipped with a Chromeleon program for the system manipulation, data acquisition and analysis, a photodiode array UV-Vis detector (Ultimate 3000 HPLC, Dionex, Thermo Fisher, Waltham, MA, USA), and a C18 HPLC column purchased from Agilent (PLRP-S C18 column, 3 μm, 150 mm × 4.6 mm, Agilent Technologies, Santa Clara, CA, USA). The mobile phase consisting of 4 mM ammonium acetate aqueous solution (A) and methanol (B) [15] was pumped at a flow rate of 0.3 mL·min^−1^. Both mobile phases were adjusted to pH 1.5 with *o*-phosphoric acid. A gradient elution was used to give an optimum separation. Details of the gradient elution program are summarized in Table 7. A total run time of 20 min was used. The injection volume was 50 µL. The separation was performed at room temperature. Orellanine was detected spectroscopically at a wavelength of 308 nm.

### 4.5. Sample Preparation for Liquid Chromatography Tandem Mass Spectrometry (LC-MS/MS) Analysis

We developed a liquid chromatography tandem mass spectrometry for only definitive confirmation of the identity of OR in tissues. The sample preparation procedure was modified from the QTOF-MS/MS procedure of analysis of OR in mushrooms [15]. Briefly, 0.1 g mouse tissue was extracted in 400 µL methanol: 3 M hydrochloric acid solution (10:1, *v*/*v*), followed by vortexing for 1 min and centrifugation at 20,000× *g* for 5 min. As in the Hermann *et al.* paper of extracting OR from mushrooms [15], we found acid was essential for extracting the OR from tissues giving better recoveries with than without addition of acid. Orellanine stock standard was prepared by weighing 2 mg of orellanine and dissolving in 2 mL of 3M HCL to create a 1000 µg/g standard. Orellanine working standards for use on LC-MS/MS were prepare by diluting the stock standard in methanol: 3M hydrochloric acid (10:1, *v*/*v*) to obtain a 1 µg/g standard.

### 4.6. LC-MS/MS Conditions

Analyses were performed using a triple-quadrupole LC-MS/MS (Varian 310 LC-MS/MS system, Agilent Technologies, Santa Clara, CA, USA) with an electrospray ionization (ESI) chamber in the positive mode, an autosampler (410 Varian Prostar autosampler, Agilent Technologies, Santa Clara, CA, USA) and two HPLC pumps (210 Varian Prostar HPLC pump, Agilent Technologies, Santa Clara, CA, USA) Needle voltage was −3000 V, collision induced dissociation gas pressure 0.20 Pa, drying gas temperature was 150 °C, nebulizer gas pressure was 3.45 × 10^5^ Pa, drying gas pressure was 6.89 × 10^4^ Pa, detector was biased at 1500 V, acquisition time was 10 min. The mobile phase consisted of solvent A: 1% formic acid, and solvent B: acetonitrile, at an isocratic elution with a ratio of 5:95, *v*/*v*) [15]. The mobile phase was modified from a paper by Hermann *et al.* [15] for use with PRP-1 column (Hamilton PRP-1 column 10 μm, 250 × 4.1 mm I.D. part# 79427, Phenomenex, Torrance, CA, USA). This column is well suited for the low pH of the extract and performs well. The flow rate was 0.4 mL/min. Orellanine was detected by LC-ESI-MRM using transition modes 253 > 163, 253 > 191, 253 > 219, 253 > 236 obtained by infusion of orellanine. Multiple transitions for this compound assist in confirmation. These transitions were similar to those used for QTOF-MS/MS procedure. This is an improvement because by expanding LC-MS/MS use to triple quadrupole for the OR compound allows greater capabilities for use of LC-MS/MS instrumentation for confirmation and quantification.

The detailed parameters of the quadrupole mass spectrometers are listed in Table 8. A 20 µL sample was injected into the column.

### 4.7. Statistical Analysis

Statistical analysis to detect differences between the control group of mice injected with 0.9% normal saline and the OR treated mice was performed using a commercially available statistics package (GraphPad Software, Inc., La Jolla, CA, USA). Endpoints measured and statistically analyzed included animal body weight; organ weights including liver, kidney, and spleen; urine volume; complete blood cell counts and differentials; and serum enzymes. A one-way analysis of variance (ANOVA) was performed followed by a Dunnett’s post-test for comparison of the means of each group for each end point to that of controls. A *p* value of less than 0.05 was considered statistically significant.

## 5. Conclusions

In conclusion, this study has moved research on diagnosis of *Cortinarius* mushroom-induced toxicity forward. We have developed definitive tissue-based analytical test methods for detection, quantitation and confirmation of OR, a biomarker of *Cortinarius* mushroom poisoning. The HPLC LOQ was 10 ppm while the LC-MS/MS LOD was 20 ppb. Mice intoxicated with OR contained an average of 17 ppm OR 96 h post OR injection. Besides the kidney, the study showed that the liver is also affected by OR. Splenic lymphocytolysis and reduced splenic weight were notable findings reported for the first time. OR-induced serum biochemical changes included elevated BUN, ALT, and phosphorus and decreased serum calcium, sodium, and albumin. Orellanine caused thrombocytopenia, while the leukogram was characterized by elevated neutrophils and significantly decreased lymphocytes and eosinophils. Collectively, these findings will be beneficial to clinicians and diagnosticians in reaching a conclusive diagnosis of OR poisoning in animals.

## Figures and Tables

**Table 1:** Organ weight changes in mice given orellanine compound with control group given 0.9% normal saline.

Organ Weights		Orellanine		
		Hours after Intoxication		
	Control (*n* = 12)	2 (*n* = 12)	24 (*n* = 12)	96 (*n* = 8)
Lung (mg)	167.4 ±33.8	129.7 ± 37.5 *	151.0 ± 21.8	139.8 ± 29.1
Spleen (mg)	66.0 ± 15.2	67.5 ± 8.9	46.6 ± 11.7	27.7 ± 10.8 ^†^
Liver (mg)	1203.3 ± 138.7	961.7 ± 342.0 *	922.8 ± 168.6 ^†^	892.3 ± 124.6 ^†^
Kidney (mg)	297.2 ± 23.9	302.9 ± 42.1	288.3 ± 26.75	273.7 ± 20.2

Note that orellanine caused a statistically significant reduction in lung, splenic, and liver weights compared to the controls. Values given are means ± 1 standard deviation. * *p* < 0.05 compared to controls; ^†^ *p* < 0.01 compared to controls.

**Table 2:** Total and differential blood cell counts in mice given orellanine compared to control group given 0.9% normal saline.

Complete Blood Count		Orellanine		
		Hours after Intoxication		
	Control (*n* = 4)	2 (*n* = 5)	24 (*n* = 5)	96 (*n* = 3)
White blood cells (10^3^/µL)	5.9 ± 2.0	3.6 ± 0.5	4.8 ± 1.3	3.8 ± 1.1
Red blood cells (10^3^/µL)	8.9 ± 1.3	8.6 ± 0.2	9.8 ± 1.0	10.3 ± 0.9
Neutrophils (10^3^/µL)	0.6 ± 0.3	0.9 ± 0.5	2.4 ± 1.4 *	2.7 ± 0.9 *
Monocytes (10^3^/µL)	0.1 ± 0.1	0.1 ± 0.1	0.1 ± 0.1	0.1 ± 0.1
Lymphocytes (10^3^/µL)	5.2 ± 1.1	2.7 ± 0.4 ^†^	2.3 ± 0.7 ^†^	1.1 ± 0.8 ^†^
Eosinophils (10^3^/µL)	0.2 ± 0.1	0.0 ± 0.0 *	0.0 ± 0.0 *	0.0 ± 0.0 *
Platelets (10^3^/µL)	1161.3 ± 110.2	989.8 ± 163.0	1092.0 ± 110.1	333.0 ± 258.8 ^†^
Hemoglobin (gm/dL)	14.0 ± 1.1	13.0 ± 0.2	14.6 ± 1.7	16.0 ± 1.0
Hematocrit (%)	48.5 ± 3.	44.9 ± 2.0	49.7 ± 5.5	48.7 ± 5.3
Mean corpuscular volume (MCV) (fL)	52.9 ± 1.0	52.5 ± 3.0	50.7 ± 1.3	47.1 ± 1.6 ^†^
Mean corpuscular hemoglobin (pg)	15.3 ± 0.21	15.2 ± 0.2	14.9 ± 0.2	15.5 ± 0.5
Mean corpuscular hemoglobin concentration (MCHC) (gm/dL)	28.9 ± 0.3	28.9 ± 1.1	29.4 ± 0.7	32.9 ± 2.1 *
Red blood cell (RBC) distribution width (%)	12.9 ± 1.1	13.0 ± 0.5	12.7 ± 0.3	12.5 ± 0.9
Mean platelet volume (fL)	4.5 ± 0.2	5.2 ± 1.1	4.4 ± 0.1	7.2 ± 3.7

Note that orellanine caused a statistically significant increase in total neutrophil count and mean corpuscular hemoglobin concentration, and reduced the total lymphocyte, eosinophil, and platelet counts. Values given are means ± 1 standard deviation. * *p* < 0.05 compared to controls; ^†^ *p* < 0.01 compared to controls.

**Table 3:** Serum chemistry profile in mice injected with orellanine compared to control group given 0.9% normal saline.

Serum Clinical Parameters		Orellanine		
		Hours after Intoxication		
	Control (*n* = 11)	2 (*n* = 11)	24 (*n* = 10)	96 (*n* = 3)
Alanine aminotransferase (IU/L)	38.1 ± 10.1	53.1 ± 20.2	62.8 ± 37.4	164.3 ± 219.0 ^†^
Blood urea nitrogen (mg/dL)	17.9 ± 5.2	16.3 ± 5.5	50.0 ± 51.2	77.3 ± 89.3 ^†^
Alkaline phosphatase (IU/L)	101.5 ± 10.3	105.3 ± 14.3	136.2 ± 37.3	183.7 ± 162.3 *
Albumin (gm/dL)	4.2 ± 0.2	3.5 ± 0.3	2.8 ± 1.0 ^†^	3.7 ± 1.6
Calcium (mg/dL)	11.3 ± 0.4	9.7 ± 1.4	7.7 ± 1.2 ^†^	10.2 ± 3.8
Glucose (mg/dL)	274.4 ± 45.1	174.1 ± 78.9	186.9 ± 120.0	352.3 ± 269.8
Phosphorus (mg/dL)	8.4 ± 1.2	10.6 ± 1.9	11.9 ± 3.9 *	8.8 ± 3.8
Potassium (mEq/L)	7.7 ± 0.5	8.1 ± 0.5	8.4 ± 0.3	8.4 ± 3.7
Sodium (mEq/L)	158.5 ± 7.1	156.5 ± 4.9	158.7 ± 7.3	137.0 ± 15.4 ^†^
Total bilirubin (mg/dL)	0.6 ± 0.8	0.3 ± 0.0	0.3 ± 0.0	0.3 ± 0.2
Creatinine (mg/dL)	0.2 ± 0.1	0.2 ± 0.0	0.4 ± 0.3	0.4 ± 0.4
Total Protein (gm/dL)	5.7 ± 0.3	4.9 ± 0.3	5.4 ± 0.9	5.9 ± 2.3

Note that orellanine caused significant elevations in serum alanine aminotransferase (ALT), blood urea nitrogen (BUN), alkaline phosphatase (ALP), and phosphorus levels. Orellanine also caused a reduction in serum albumin, calcium, and sodium. Values given are means ± standard deviation. * *p* < 0.05 compared to controls; ^†^ *p* < 0.01 compared to controls.

**Table 4:** A summary of calibration curves established by fortified kidney tissues, range, regression equation, and linearity using HPLC *.

Run	Fortification Levels, µg/g	Equation	Linearity (R^2^)
1	15, 20, 30, 40, 50	*y* = 1.81*x* − 7.15	0.97
2	10, 15, 20, 25, 30, 35, 40, 50	*y* = 1.85*x* − 3.72	0.99
3	10, 15, 20, 25, 30, 35, 40, 50	*y* = 1.85*x* − 1.78	0.99

* High performance liquid chromatography.

**Table 5:** A summary of inter-run repeatability (RSD *) and accuracy of fortified kidney tissues using HPLC ^†^.

Fortification Levels, µg/g	RSD *, %	Average Nominal Concentration ^‡^, µg/g	Accuracy ^§^, %
15.0	6.4	14.5	3.3
20.0	9.8	18.6	7.1
30.0	5.6	31.6	5.2
40.0	1.3	40.8	2.1
50.0	2.3	49.2	1.5

* Relative standard deviation. It is based on three analyses. ^†^ High performance liquid chromatography. ^‡^ Nominal concentration is back-calculated from the calibration curve using the response of peak area from HPLC analysis. The average nominal concentration is calculated based on three analyses for each fortification level. ^§^ Accuracy is calculated as (Average nominal concentration-fortification level)/fortification level × 100%. It indicates the closeness of the concentration quantified by the calibration curve to the fortification level.

**Table 6:** A summary of orellanine kidney concentrations by HPLC in given orellanine and euthanized over different times (2 h, 24 h, and 96 h post exposure). The results are reported in the format of “mean ± standard deviation”.

Time after Exposure, h	Orellanine Concentration, µg/g
2	97 ± 51
24	45 ± 34
96	17 ± 1
Control	Below LOQ (<10 µg/g)

*n* = 6 for each time intervals (2 h, 24 h, and 96 h) and the control group.

**Table 7:** A summary of HPLC * gradient for orellanine analysis.

Time, min	4 mM Ammonium Acetate Aqueous Solution (A), %	Methanol (B), %
0–2.0	98	2
2.0–4.0	15	85
4.0–20.0	15	85

* High performance liquid chromatography.

**Table 8:** A summary of LC-ESI-MRM * conditions for orellanine analysis.

Q1, *m/z*	Q3, *m/z*	Capillary Voltage, V	Collision Voltage, V	Dwell Time, ms
253	163	36	20	0.5
253	191	36	25.5	0.5
253	219	36	20	0.5
253	236	36	14	0.5

* Liquid chromatography-triple quad mass spectrometry, electrospray ionization, multiple reaction monitoring.
